# Environmental variability drives functional plasticity in the gill-associated microbiome of *Lithodes santolla*: a meta-transcriptomic perspective

**DOI:** 10.1186/s40168-025-02215-6

**Published:** 2025-10-01

**Authors:** Alexandra Brante, Paulina Bustos, Claudio Ortega-Muñoz, Eliana Paola Acuña Gómez, Vicenzo Brante, Rodolfo Farlora

**Affiliations:** 1 Programa de Magíster en Ciencias Biológicas Mención Biodiversidad y Conservación, Instituto de Biología, Facultad de Ciencias, Universidad de Valparaíso, Valparaíso, Chile; 2 Centro de Estudios del Cuaternario de Fuego — Patagonia y Antártica (CEQUA), Punta Arenas, Chile; 3 Laboratorio de Microbiología Integrativa e Innovación Tecnológica (MIIB-Lab), Facultad de Ciencias, Instituto de Biología, Universidad de Valparaíso, Valparaíso, Chile; 4 Centro de Investigación y Gestión de Recursos Naturales (CIGREN), Universidad de Valparaíso, Valparaíso, Chile; 5 Laboratorio de Biotecnología Acuática y Genómica Reproductiva (LABYGER), Instituto de Biología, Facultad de Ciencias, Universidad de Valparaíso, 2360102 Valparaíso, Chile

**Keywords:** *Lithodes santolla*, Metatranscriptomic, Gill-associated microbiome, Host-microbe interactions, Sub-Antarctic environment

## Abstract

**Background:**

Environmental variability shapes microbial community composition and function, yet its influence on microbial gene expression and host-microbiome interactions in sub-Antarctic regions remains poorly understood. Gills serve as the primary interface between aquatic organisms and their environment, harboring diverse and dynamic microbial communities that play a fundamental role in host physiology. Using a metatranscriptomic approach, this research aims to explore the influence of abiotic fluctuations in Patagonian fjords on the functional profile of the gill-associated microbiome in the southern king crab (*Lithodes santolla*) holobiont. By assessing shifts in microbial composition and gene expression, this research aims to uncover functional pathways linked to microbial metabolic adjustments and the host’s resilience. The findings provide insights into microbiome-driven functional responses in marine species and may inform conservation strategies under environmental change.

**Results:**

Microbial gene expression profiles from individuals collected at two environmentally distinct locations, Ballena Sound and Choiseul Bay, revealed slight differences in microbial composition, with Proteobacteria dominating at both sites. Functional annotation identified key metabolic pathways involved in energy production, stress response, and microbial interactions, highlighting distinct adaptive mechanisms to environmental fluctuations. Differential expression analysis revealed shifts in carbon fixation, ion transport, and oxidative stress responses, suggesting that these physiological responses could be modeled by environmental conditions. Additionally, host-associated transcripts showed differential enrichment in immune regulation and metabolic homeostasis pathways, suggesting microbiome-mediated effects on host physiology.

**Conclusions:**

These findings offer first insights into the dynamic relationship between environmental factors and microbial functionality in *L. santolla*, highlighting the significance of gill-associated microbiome plasticity in adapting to changing habitats. These results improve our understanding of microbiome-driven functional responses to sub-Antarctic environments, offering valuable perspectives for assessing holobiont resilience in these fluctuating ecosystems.

Video Abstract

**Supplementary Information:**

The online version contains supplementary material available at 10.1186/s40168-025-02215-6.

## Introduction

Multicellular organisms harbor diverse microbial communities, including archaea, bacteria, fungi, protists, and viruses, which inhabit their external and internal surfaces [1, 2]. These microbial communities, collectively known as the microbiome, play a fundamental role in host physiology by contributing to metabolic regulation, immune response, and adaptation to environmental changes [3, 4]. The microbiome helps maintain homeostasis through complex interactions, providing benefits such as nutrient cycling, protection against pathogens, and enhancement of stress tolerance [5]. The holobiont, which refers to the ecological and functional unit formed by the host and its associated microbiome, relies on these symbiotic interactions to maintain key physiological processes [6, 7]. However, the composition and functionality of the microbiome are not static; they fluctuate in response to environmental conditions such as temperature variations, salinity shifts, pollution, and oxygen availability [1]. These changes can alter microbial diversity and functionality, potentially disrupting host-microbiome interactions and affecting overall host health [8].

Marine organisms are particularly susceptible to environmental variations due to their direct exposure to abiotic stressors and fluctuating conditions [9]. Therefore, microbial communities’ taxonomic composition and functional dynamics may vary depending on the environmental conditions their hosts experience [10–12]. Among these microbial assemblies, the gill microbiome is particularly interesting due to its role as a primary interface between the host and its surrounding environment [13, 14]. As a key respiratory and excretory organ, the gills facilitate gas exchange, nitrogen metabolism, detoxification, and immune defense [13]. Thus, changes in the composition and function of the gill microbiome could directly impact host physiology [15]. Since gill-associated microbial communities are continuously exposed to external environmental conditions, they serve as an ideal model for studying microbiome responses to environmental variability.

The southern king crab (*Lithodes santolla*) is a benthic decapod of ecological and economic interest in the Southwest Atlantic and Southeast Pacific oceans [16]. This species inhabits deep cold waters along coastal and fjord areas in sub-Antarctic regions with pronounced salinity fluctuations, low temperatures, and seasonal variations in oxygen availability [17–19]. The large range of abiotic conditions *L. santolla* is exposed to could lead to shifts in microbial diversity, affecting microbial taxa’s relative abundance and function, as described in other organisms [20]. In marine crustaceans, environmental stressors have been shown to alter the balance between beneficial and opportunistic microbes, potentially disrupting essential symbiotic interactions [21–23]. Gills represent a critical interface between the host and the environment; therefore, their microbiome is particularly susceptible to environmental fluctuations. Despite increasing evidence that environmental variability influences marine host microbiome composition [4, 24], our understanding of how these changes impact microbial function in *L. santolla* remains nonexistent. Understanding how microbial communities adjust their composition and functional activity under fluctuating conditions is crucial for elucidating the mechanisms underlying host-microbiome interactions in marine crustaceans.

To address this gap, metatranscriptomics offers a powerful approach by capturing real-time gene expression profiles, allowing researchers to identify functional responses of microbial communities to environmental changes [25–27]. It provides insights into the composition of microbial communities through the analysis of transcripts and the identification of differentially expressed genes in response to variations in environmental conditions [28, 29]. Metatranscriptomic analyses of gill-associated microbiomes in multiple marine species have provided insights into functional adjustments to environmental variability. Studies on mollusks have revealed significant changes in microbial gene expression during acclimatization to new environmental conditions, particularly in pathways related to oxidative phosphorylation and stress response, as observed during a transition from hydrothermal vent to sea-level conditions [30]. Similarly, gill microbiome composition in shallow subtidal and intertidal crab species has been observed to vary based on respiratory adaptations, suggesting a functional link between microbial activity and host physiology [31].

Building on these findings, this study aims to evaluate the impact of environmental variability on the functional profile of the gill microbiome of *L. santolla*, identifying potential functional responses and characterizing host-microbiota dynamics under two environmental conditions. Specifically, we seek to determine how microbial composition and gene expression shift in response to abiotic fluctuations, focusing on functional pathways related to host resilience, metabolic adjustments, and microbial community stability.

## Materials and methods

### Sample collection, RNA extraction, and sequencing

Sampling took place in March 2024 at Ballena Sound (53°40′30″ S, 72°37′39″ W) and Choiseul Bay (53°45′34″ S, 72°16′05″ W). The sites are approximately 32 km apart in a straight line, both located in the Santa Inés Island (Fig. S1). Although connected by a narrow channel, both areas exhibit semi-enclosed conditions due to geographic constrictions and bathymetric features that limit water exchange. Ten southern king crab (*L. santolla*) individuals, five from each site, were collected, sexed, and measured for carapace length (CL), carapace width (CW), and weight (Table S1). During sampling, environmental parameters—including salinity, temperature, pH, dissolved oxygen, and oxygen saturation—were recorded using an WiMo multiparameter probe (nke Instrumentation). Gill tissues were excised from each specimen, preserved in RNAlater™ solution (Thermo Fisher Scientific), and transported to the laboratory, where they were stored at − 80 °C. Total RNA was extracted from gill tissue using TRIzol® reagent (Thermo Fisher Scientific) according to the manufacturer’s instructions. RNA quantity was measured with a NanoDrop Lite Spectrophotometer (Thermo Fisher Scientific), while RNA quality was evaluated using a Qubit™ RNA IQ Assay Kit in a Qubit® 4 Fluorometer (Thermo Fisher Scientific). The extracted RNA was then freeze-dried and shipped to Novogene (Sacramento, USA) for sequencing. Upon reconstitution, 1 μg of total RNA was used for each library preparation. To retrieve the holobiont transcriptomic data of both host gills and their associated microbiome (including bacteria, archea, protists and fungi), 10 cDNA libraries were prepared using rRNA ribodepletion (Ribo-Zero Plus rRNA Depletion Kit, Illumina). NEBNext Ultra II RNA Library Prep Nondirectional library (New England Biolabs) was employed, and libraries were sequenced on an Illumina NovaSeq 6000 platform using paired-end reads (2 × 150 bp length). The raw reads were uploaded to the NCBI’s SRA database (BioProject PRJNA1254097).

### De novo* transcriptome assembly*

Quality control assessment was conducted using the FastQC tool (http://www.bioinformatics.babraham.ac.uk/projects/fastqc/). Adapters and low-quality sequences were filtered using Trimmomatic (v0.39) [32] with the following settings: The adapter sequences TruSeq3-PE-2.fa were removed using the ILLUMINACLIP tool with the following settings: LEADING:5, TRAILING:5, SLIDINGWINDOW:4:20, and MINLEN:150. The trimmed reads were then reimported to FastQC for a comprehensive assessment of the overall trimming quality. SortMeRNA (v4.3.6) [33] was employed to sort and remove rRNA reads, utilizing the SILVA and Rfam eukaryotic, prokaryotic, and virus databases. Without a reference genome for *L. santolla*, de novo transcriptome assembly was conducted using Trinity RNA-seq (v2.15.1) [34] with a minimum contig length of 150 bp.

### Assembly quality and completeness

The mapping alignment rate was evaluated using Bowtie2 (v 2.5.4) [35] under the default settings to evaluate the proportion of retained sequences. Clustering was performed with v4.8.1 [36] to reduce the transcript redundancy using a 95% sequence identity threshold and a word size of 10. Furthermore, assemblies’ completeness was evaluated using Benchmarking Universal Single-Copy Orthologs (BUSCO, v5.8.2) [37], which employed arthropod_odb10, eukaryote_odb10, and prokaryote (bacteria_odb12 and archaea_odb12) datasets. The percentage of complete, fragmented, and missing orthologs was recorded to determine the coverage of assemblies relative to the expected gene content in those lineages.

### Taxonomic and functional annotation

Open reading frames (ORFs) were predicted with TransDecoder (v5.7.1), with a minimum length of 50 amino acids (AA) to perform a taxonomic and functional annotation. Sequence similarity searches were conducted in DIAMOND [38] against the NCBI nonredundant (NR) protein database (downloaded on November 2, 2024) with an *E*-value cutoff of 1E-5 and the *more-sensitive* mode. Assigned ORFs were imported to MEGAN6 Community-Edition (v6.25.10) [39] based on the lowest common ancestor (LCA) criteria. Host-derived ORFs were classified as arthropod sequences, while bacteria, archaea, fungi, and protists were designated as microbiome-derived sequences. Final taxonomic assignments were determined using a custom in-house script, which extracted the complete taxonomic lineage of each ORF by parsing NCBI taxonomic identifiers (TaxIds) retrieved from DIAMOND BLASTp hits. This script cross-referenced TaxIds with the NCBI taxonomy database, ensuring accurate hierarchical classification from domain to species level. Microbial diversity was further evaluated using Bray–Curtis dissimilarity to compare taxonomic composition, and chi-square tests were applied to assess whether microbial taxa were significantly different between Ballena and Choiseul. As chi-square requires at least 80% of the data to have expected values greater than 5, taxa with lower counts were removed before analysis [40]. Moreover, functional annotation and ortholog-based classification for both host and microbiome-derived sequences were performed using the command-line version of eggNOG-mapper (v2) [41] retrieving Gene Ontology terms, KEGG pathways, COG categories, and Enzyme Commission numbers. Additionally, KAAS (KEGG Automatic Annotation Server) was employed to obtain enhanced KEGG pathway annotation. The ggplot2, Circos, and networkD3 packages were used for plotting data output in R (v4.4.1).

### Differential expression and functional enrichment analysis

Transcript mapped-based quantification was performed using the Salmon tool [42] within the Trinity package with the *align_and_estimate_abundance.pl* script. The resulting abundance estimates were merged into raw counts and normalized using the trimmed mean of M values (TMM) method [43], as implemented in the *abundance_estimates_to_matrix.pl* script in Trinity. Furthermore, differential expression analysis was conducted using the bioconductor package DESeq2 [44] for statistical analysis of pairwise comparisons between conditions. The following parameters were employed to minimize false positives: false discovery rate (FDR) ≤ 0.05 and a log₂ fold change ≥ 2. Only transcripts with padj < 0.05 and detected in at least two biological replicates per condition were retained. Furthermore, to explore sample relationships and variance, correlation analyses were performed using the *PtR.pl* script in Trinity, which includes both sample-to-sample correlation matrices and principal component analysis (PCA). Likewise, hierarchical clustering of differentially expressed genes (DEGs) was conducted using the *analyze_diff_expr.pl* script in Trinity. Functional enrichment analysis was conducted on DEGs using Gene Ontology (GO) term enrichment and KEGG pathway analysis. The GO term enrichment analysis was performed with GOSeq, using GO terms retrieved from the prior functional annotation and *run_GOSeq.pl* script in Trinity. A network representation of the top enriched GO terms was constructed to visualize functional relationships, selecting terms based on their false discovery rate (FDR) values. Similarly, KEGG pathway enrichment analysis was carried out separately on DEGs using clusterProfiler in R, enabling the identification of significantly enriched metabolic and signaling pathways.

## Results

### Environmental characteristics of sampling sites

Southern king crab (*L. santolla*) individuals were obtained from two distinct locations: Ballena Sound and Choiseul Bay, both located in Punta Arenas, Chile. During the sampling period, environmental measurements revealed that Ballena Sound had an average salinity of 24.66 practical salinity units (PSU), reflecting its greater exposure to glacial inputs and associated hydrological fluctuations. In contrast, Choiseul Bay showed slightly elevated salinity at 29.21 PSU. Additionally, Choiseul Bay had comparatively higher temperatures (8.74 °C), dissolved oxygen concentrations (11.17 mg/L), and oxygen saturations (95.36%) than Ballena Sound, which had temperatures of 7.63 °C, dissolved oxygen concentrations of 9.86 mg/L, and oxygen saturations of 92.31%, respectively (Table S2).

### De novo* gill holo-transcriptome assembly*

The sequencing of five biological replicates per location from gill tissue of *L. santolla* resulted in 10 cDNA libraries. A total of 332,804,332 clean reads were obtained after quality assessment, adapter removal, and rRNA filtering (Table S3). Three holo-transcriptomes are as follows: (i) Global (Ballena + Choiseul), (ii) Ballena, and (iii) Choiseul were assembled to assess microbial composition and host-microbiome functionality between populations (Table 1).

**Table 1:** Gills de novo holobiont transcriptome statistics after redundance removal

Metrics	Global assembly	Ballena assembly	Choiseul assembly
Number of transcripts	1,318,250	816,784	932,503
Number of Trinity “genes”	1,087,854	681,424	776,789
Total bp in assembly	582,147,838	369,495,185	425,077,194
Max contig length	39,718	39,169	37,975
Min contig length	128	129	129
Average contig length (bp)	441.6	452.4	455.8
Median contig length (bp)	297	302	299
%GC	40.46	40.40	40.57
N20 contig length	1370	1439	1520
N50 contig length	497	514	522
Number of contigs in N50	282,479	171,878	190,780
Number of transcripts over 1000 bp	83,887	54,505	65,803
Mapping rate to reference transcriptome	92.9%	92.6%	92.0%

Completeness assessment using BUSCO, based on eukaryotes, arthropods, and prokaryotes orthologs, revealed a high representation of conserved genes. The assemblies shared over 90% of eukaryotic and arthropod orthologs and over 60% of prokaryotic orthologs (Table S4). For the 255 eukaryotic orthologs, the global assembly showed the highest completeness (99.2%), with 66.3% as single copies and 32.9% as duplicates. The Ballena assembly had 96.9% completeness (71.4% single copy, 25.5% duplicates), while Choiseul reached 96.1% (74.5% single copy, 21.6% duplicates). For the 1013 arthropod orthologs, the global assembly showed 94.1% completeness (63.9% single copy, 30.2% duplicates). Choiseul had 92.6% completeness (69.0% single copy, 23.6% duplicates), while Ballena reached 92.5% (70.0% single copy, 22.5% duplicates). Finally, for the 311 prokaryotic orthologs, the global assembly had 65.3% completeness (17.7% single copy, 47.6% duplicates). Choiseul showed 63.4% completeness (17.4% single copy, 46.0% duplicates), and Ballena reached 63.0% (22.8% single copy, 40.2% duplicates).

**Fig. 1:**
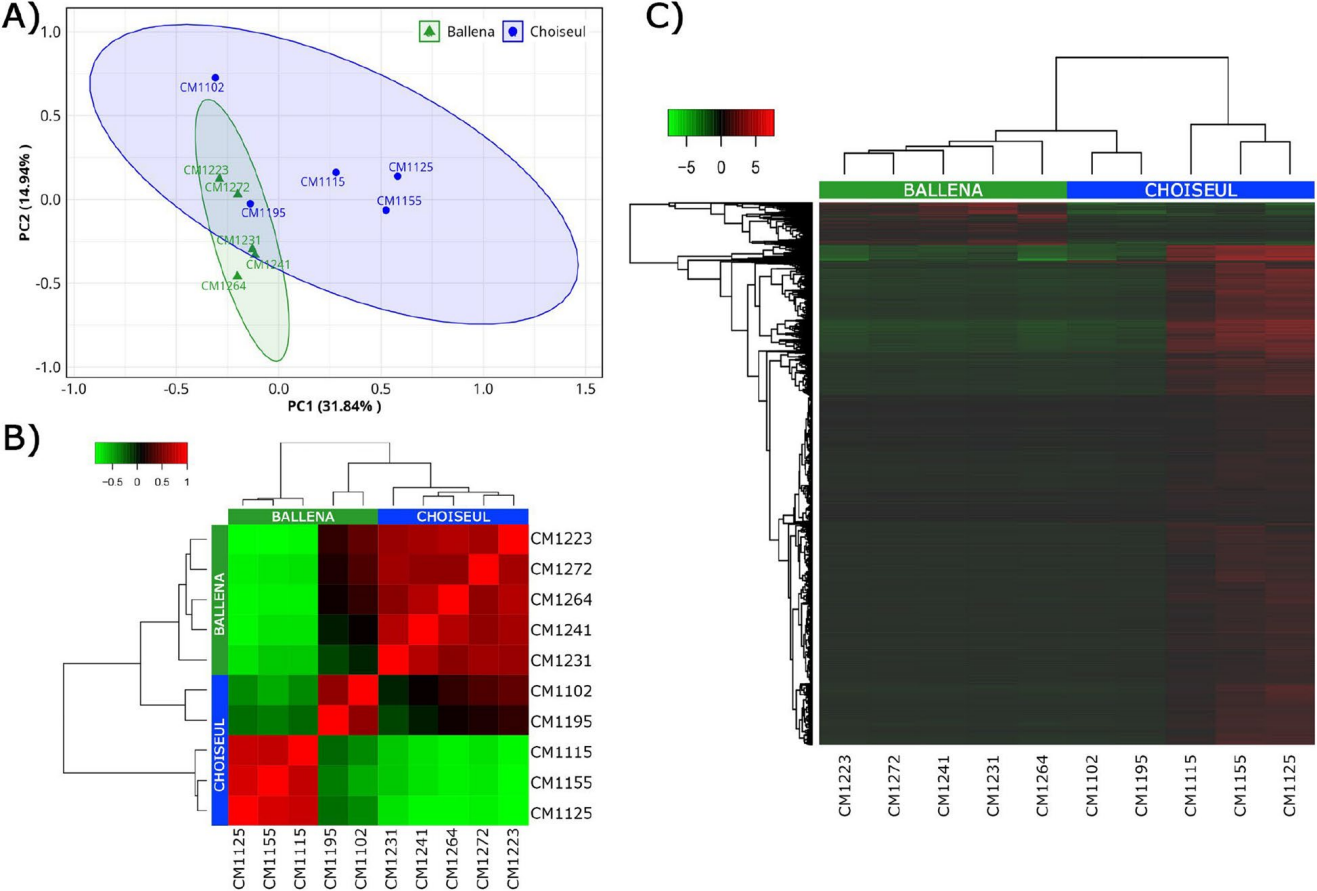
Transcriptomic variation and differential expression patterns between Ballena and Choiseul samples. **A** PCA plot showing the distribution of samples based on gene expression profiles. Each point represents an individual sample, grouped by locality (Ballena in green triangles, Choiseul in blue dots). Ellipses indicate 95% confidence intervals for each group. **B** Sample-to-sample correlation heat map based on Pearson’s correlation coefficients. Samples cluster according to their locality, reflecting overall transcriptional similarity within sites. **C** Hierarchical clustering heat map of DEGs between Ballena and Choiseul. Rows represent DEGs, and columns represent samples. Color gradients indicate relative expression levels (red, upregulated; green, downregulated)

To assess transcriptomic variation across samples, a principal component analysis (PCA) was conducted using RNA-seq data. Principal components PC1 and PC2 accounted for 46.78% of the observed variance (Fig. 1A). However, two samples from Choiseul clustered closer to the Ballena group, suggesting overlapping in their transcriptional profiles. This inter-individual variation was also reflected in the hierarchical clustering, where the same two Choiseul individuals grouped closer to the Ballena cluster (Fig. 1B). Differential expression analysis identified a total of 7819 DEGs between the groups, of which 7207 were upregulated in Choiseul and 612 were upregulated in Ballena (Fig. 1C).

### Taxonomic composition of the microbiome in the L. santolla holobiont

Sequences from de novo holo-transcriptomic assemblies were annotated using the NCBI nonredundant (NR) database. The Ballena assembly contained 68,115 assigned ORFs (40,408 host; 14,565 microbial, and 13,142 other taxa), while Choiseul had 78,513 assigned ORFs (41,369 host; 26,825 microbial, and 10,319 other taxa). Microbial ORFs were classified into archaea, bacteria, fungi, and protists. In Ballena, 32 (0.2%) archaeal, 9763 (67.0%) bacterial, 1594 (11.0%) fungal, and 3176 (21.8%) protist ORFs were identified, while in Choiseul, 56 (0.2%) archaeal, 17,496 (65.2%) bacterial, 1976 (7.4%) fungal, and 7297 (27.2%) protist ORFs were detected.

**Fig. 2:**
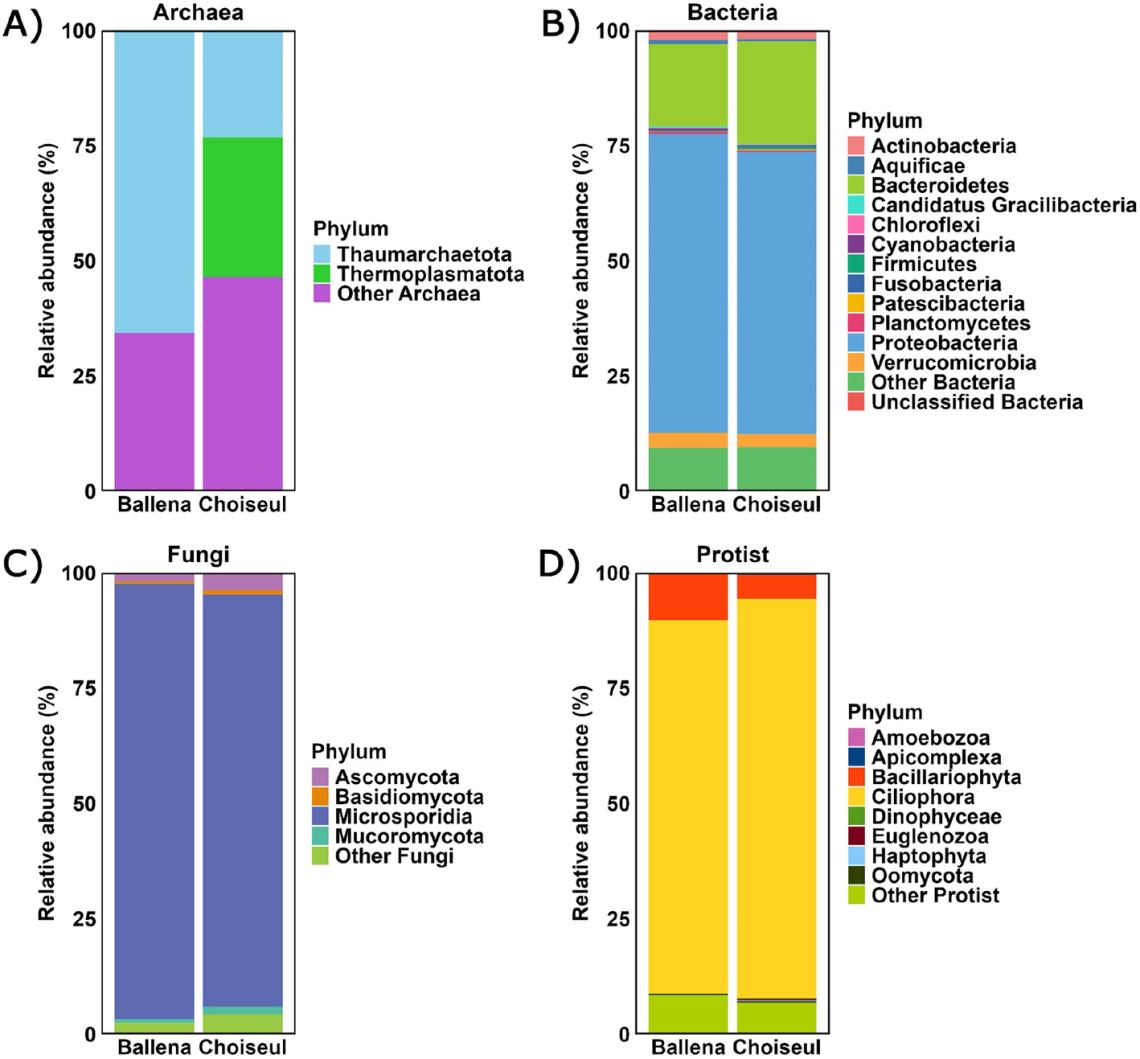
Relative abundance of microbial phyla associated with the gills of *L. santolla* from Ballena Sound and Choiseul Bay. Taxonomic profiles are shown for **A** Archaea, **B** Bacteria, **C** Fungi, and **D** Protists. Bars represent the average relative abundance per site; low-abundance taxa are grouped as “other”

Archaeal phyla were less represented overall. Thaumarchaeota was the predominant phylum in Ballena, accounting for 0.1% of microbial ORFs (21 ORFs), while in Choiseul, Thermoplasmatota represented 0.06% (17 ORFs) (Fig. 2A). Among bacteria, Proteobacteria was the dominant phylum in both sites, accounting for 43.5% in Ballena (6,336 ORFs) and 40.0% in Choiseul (10,711 ORFs). This was followed by Bacteroidetes, which comprised 12.0% in Ballena (1745 ORFs) and 14.5% in Choiseul (3,890 ORFs), and Verrucomicrobia, representing 2.2% in Ballena (316 ORFs) and 1.8% in Choiseul (490 ORFs) (Fig. 2B).

Among fungi, Microsporidia was the most abundant phylum, representing 10.3% of microbial ORFs in Ballena (1505 ORFs) and 6.6% in Choiseul (1766 ORFs) (Fig. 2C). Regarding protists, Ciliophora was the dominant protist phylum, comprising 17.7% of microbial ORFs in Ballena (2572 ORFs) and 18.5% in Choiseul (6314 ORFs). Bacillariophyta accounted for 2.2% in Ballena (317 ORFs) and 1.1% of microbial ORFs in Choiseul (379 ORFs) (Fig. 2D).

Moreover, comparative analysis of microbial composition between Ballena and Choiseul revealed differences in community structure. Bray–Curtis dissimilarity analysis showed a slight difference in microbial community and bacterial composition between conditions (Bray–Curtis = 0.297). Additionally, the chi-square test for taxonomic distribution differences was highly significant (*χ*^2^ = 549.60, *p* < 7.64E-97), indicating that microbial composition varies between conditions.

### Functional analysis of L. santolla holobiont

Gene Ontology (GO) term analysis was performed to characterize the general functional categories associated with *L. santolla* and its microbiome from Ballena and Choiseul locations. GO terms were classified into three main categories: Biological process (BP; GO:0008152), cellular component (CC; GO:0005575), and molecular function (MF; GO:0003674) (Fig. S3A). A total of 26,876 genes were annotated from the Ballena assembly, comprising 22,303 host-derived genes and 2290 microbiome-derived genes. In the Choiseul assembly, 31,538 genes were annotated, including 22,711 host-derived genes and 4374 microbiome-derived genes. The number of GO terms per category differed between host and microbiome datasets. In the BP category, the Choiseul host contained the highest number of terms (13,999), followed closely by the Ballena host (13,881) (Table S5).

In contrast, microbiome datasets contained fewer terms—7863 in the CH microbiome and 6175 in the Ballena microbiome. In the CC category, the highest number of terms was recorded for the Choiseul host (1782), followed closely by the Ballena host (1757). Microbiome datasets contained fewer CC terms, with 1127 in the Choiseul microbiome and 913 in the Ballena microbiome. In the MF category, the Choiseul host contained 3611 terms and the Ballena host 3558, while microbiome datasets had 1902 terms in the Choiseul microbiome and 1435 in the Ballena microbiome. A total of 5292 BP terms, 775 CC terms, and 1128 MF terms were shared across all datasets (Fig. S3B, C, D). Overall, host datasets contained the most unique annotated functions, with the Choiseul host exhibiting the highest number of terms across all three GO categories. Likewise, among the microbiome datasets, the Choiseul microbiome showed a higher number of unique GO terms compared to the BA microbiome.

**Fig. 3:**
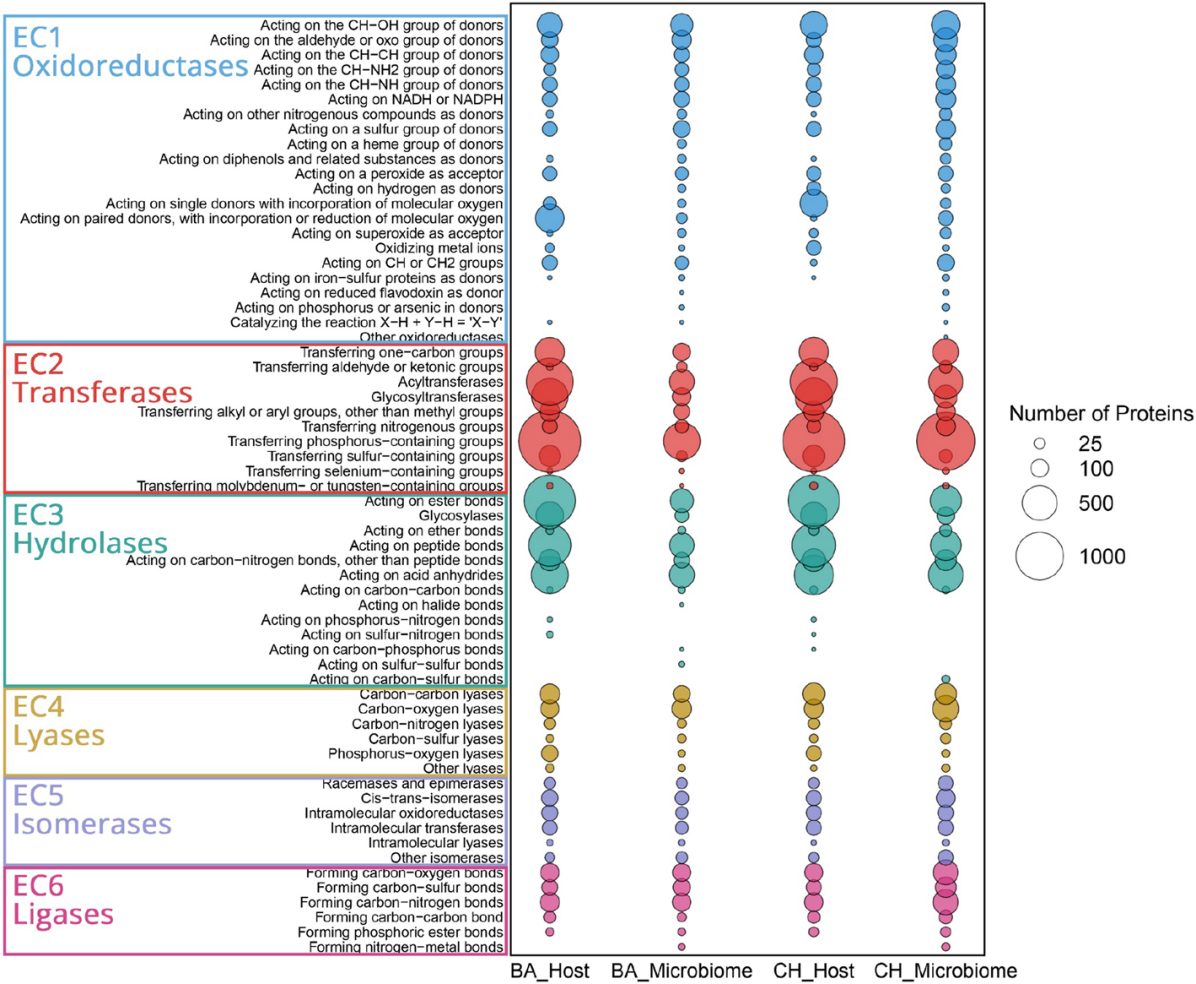
Distribution of enzyme classes (EC numbers) in *L. santolla* and its associated microbiota under Ballena Sound (BA) and Choiseul Bay (CH) conditions. The size of the bubbles corresponds to the number of proteins assigned to each enzyme category, with larger bubbles indicating a higher number of detected proteins

To further characterize the functional profiles of both conditions, enzyme commission (EC) classification analysis was performed. A total of 29,804 proteins were annotated across 3317 EC numbers. Proteins were assigned to six main classes: oxidoreductases (16.0%), transferases (40.6%), hydrolases (28.8%), ligases (5.8%), isomerases (3.7%), and lyases (5.1%) (Fig. 3). Transferases were the most abundant class, with phosphorus-containing group transferases representing 19.1% of all annotated enzymes, primarily in Choiseul host (5.9%) and Ballena host (6.0%). Hydrolases accounted for 28.8% of the dataset, acting on the ester bonds subclass covering 10.0%, most frequently in the Choiseul host (4.3%) and Ballena host (4.2%). Oxidoreductases constituted 16.0% of the dataset, with enzymes acting on the CH-OH group of donors contributing 3.4%, followed by those acting on aldehyde or oxo groups (1.6%). Carbon–oxygen dominated lyases (2.1%), primarily in the Choiseul microbiome (2.5%). Among isomerases, intramolecular oxidoreductases comprised 0.85%, while ligases, mainly involved in carbon–nitrogen bond formation, accounted for 2.0%, with the Choiseul microbiome exhibiting the highest proportion (1.8%). Overall, EC numbers varied across samples, with Choiseul host and microbiome datasets exhibiting a higher repertoire of proteins annotated than Ballena. In addition, the distribution of EC numbers varied across datasets, with the Choiseul microbiome exhibiting the highest proportion of unique ECs (292, 15.1%), followed by the Ballena microbiome (112, 5.8%), Choiseul host (41, 2.1%), and Ballena host (24, 1.2%). Four-hundred thirty-six EC numbers (22.5%) were identified as shared among all datasets (Fig. S4, Table S6). Specifically, the Choiseul host and microbiome shared 4 EC numbers (0.2%), while the Ballena host and microbiome did not share any. Conversely, the microbiome datasets shared 298 EC numbers (15.4%), whereas the host datasets exhibited the highest number of shared ECs, with 493 (25.4%).

### Functional enrichment analysis of DEGs

**Fig. 4:**
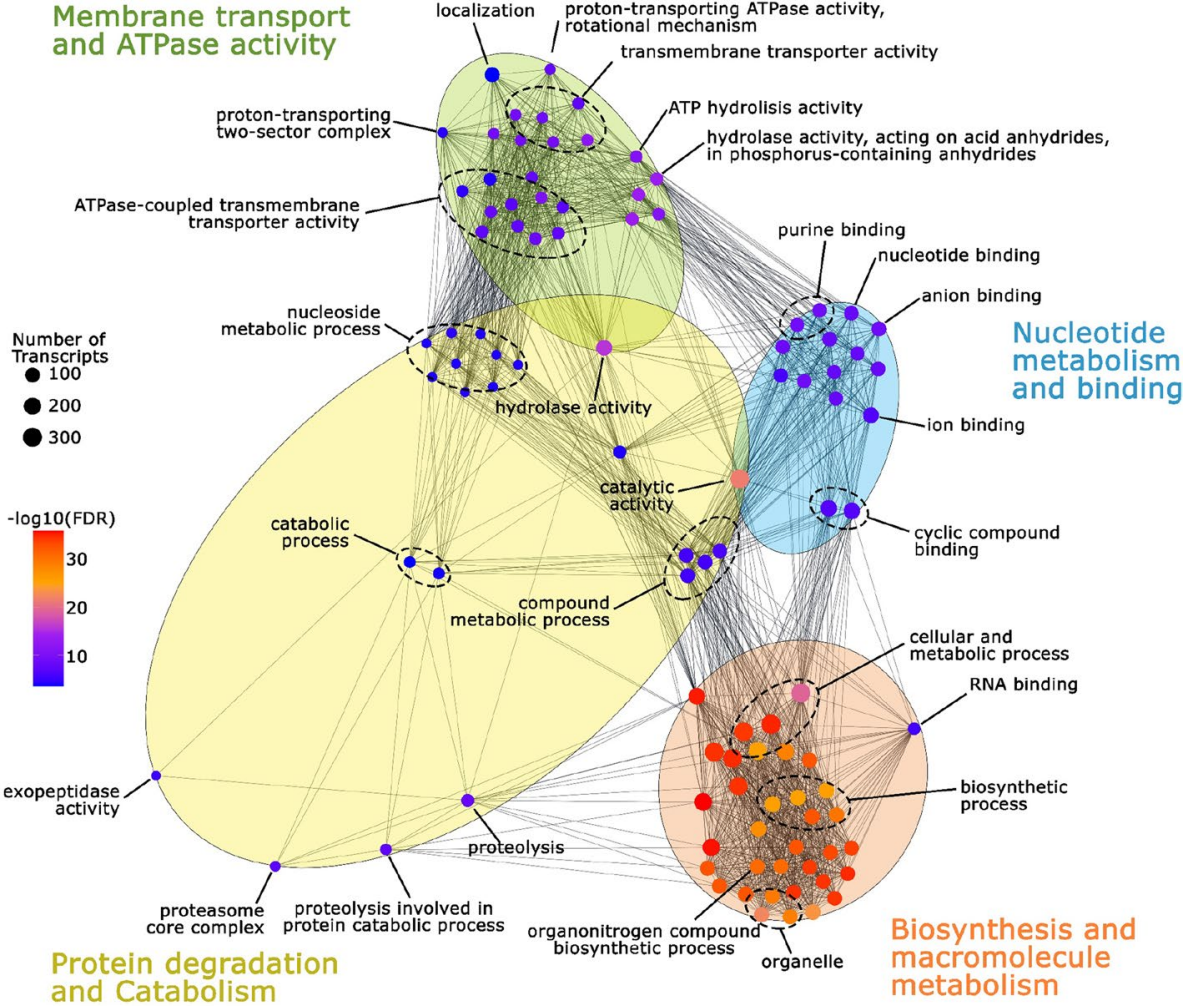
Functional network of enriched Gene Ontology (GO) terms in *L. santolla* holobiont. The node size corresponds to the number of associated transcripts, while the color gradient (red to blue) indicates the false discovery rate (FDR), with red representing the most significant terms. Functionally related GO terms are grouped into clusters

Functional enrichment analysis was performed to identify differentially enriched GO terms between Ballena and Choiseul comparison (Fig. 4, Table S5). The GO terms were clustered in four main groups, namely membrane transport and ATPase activity, nucleotide metabolism and binding, biosynthesis and macromolecule metabolism, and protein degradation and catabolism. The membrane transport and ATPase activity cluster contained the highest number of enriched terms related to transmembrane transport and ion exchange. The nucleotide metabolism and binding cluster was associated with molecular and ion binding. Biosynthesis and macromolecule metabolism included terms related to protein metabolic processes, whereas protein degradation and catabolism included proteolysis-related processes and metabolic degradation pathways.

At the microbiome level, Ballena samples exhibited enrichment in terms such as “carbon fixation” (GO:0015977), which included the upregulated gene *RuBisCO* (fold change 3.84), while “nickel cation binding” (GO:0016151) featured the *nickel-dependent hydrogenase large subunit* (fold change 8.77). Other enriched terms included “oxidoreductase activity” (GO:0016491) and “sulfur compound binding” (GO:1901681), with upregulated genes such as *ferredoxin oxidoreductase* (fold change 6.52) and *carbon monoxide dehydrogenase* (fold change 5.97). Meanwhile, Choiseul samples exhibited enriched categories such as “active monoatomic ion transmembrane transporter activity” (GO:0022853) and “catalytic activity” (GO:0003824) included the upregulated genes *F₀F₁ ATP synthase subunit alpha* (fold change 5.28), *vacuolar proton-inorganic pyrophosphatase* (fold-change 6.18), and *Na,H/K antiporter P-type* (fold change 6.15). Likewise, “ABC-type transporter activity” (GO:0140359) featured overexpressed genes involved in solute import/export activity.

On the other hand, at the host level, Ballena samples showed enrichment of GO terms such as “enzyme inhibitor/regulator” (GO:0004857/GO:0030234), both of which included the upregulated genes *crustin* (fold change 2.36) and *type IIa crustin (CruIIa-4)* (fold change 2.00), as well as “phosphate ion transport” (GO:0006817) and “monoatomic ion transport” (GO:0006811), encompassing upregulated genes such as *sodium-dependent phosphate transporter 2* (fold change 2.45) and *zinc transporter 2* (fold change 23.0). As for Choiseul samples, enrichment in terms such as “monoatomic cation transmembrane transport” (GO:0098655) and “transmembrane transport” (GO:0055085) was observed, including the upregulated genes *ammonium transporter Rh type A (RhAG)* (fold change 3.53) and *ATP synthase* (fold change 5.30). Additionally, enrichment in “antioxidant activity” (GO:0016209), “catalytic activity” (GO:0003824), and other metabolic processes revealed upregulation in genes such as *peroxiredoxin* (fold change 5.53), *thioredoxin-dependent peroxide reductase* (fold change 6.04), *fumarate hydratase* (fold change 7.48), and *NADH-ubiquinone oxidoreductase* (fold change 4.74).

Enrichment analysis on DEGs revealed enriched KEGG pathways (*FDR* ≤ 0.05) (Fig. S5, Table S7). At the microbiome level, Ballena samples showed enrichment in metabolic pathways, particularly those associated with carbon metabolism, citrate cycle (TCA cycle), glycolysis/gluconeogenesis, and oxidative phosphorylation. These pathways reflect enhanced energy production and carbon processing functions in this locality. In contrast, Choiseul microbiome samples exhibited enrichment in pathways related to protein turnover and cellular homeostasis, including the proteasome, protein processing in the endoplasmic reticulum, and autophagy. These findings suggest an increased demand for protein folding, degradation, and cellular recycling processes in Choiseul. On the other hand, at the host level, Ballena samples showed enrichment in antigen processing and presentation and endocytosis. In contrast, Choiseul samples showed enrichment in various signaling pathways, proteasome, drug metabolism (other enzymes), phagosome, and endocytosis.

## Discussion

This study comprises the first taxonomic composition and functional dynamics characterization of the gill-associated microbiome of southern king crab (*L. santolla*). Our metatranscriptomic analyses of *L. santolla* holobiont revealed differences in microbial community structure and functional activity, suggesting that environmental variability influences microbial dynamics. Multivariate analyses showed a separation between groups, but some inter-individual variability was also observed, particularly within the Choiseul group. This could be attributed to individual physiological responses or even differences in genetic backgrounds which we cannot rule out given the lack of genomic data. Although connected by a narrow channel, both areas are semi-enclosed and experience limited water exchange. Under natural temperature conditions, the 60- to 70-day-long pelagic phase of *L. santolla* [45, 46] may allow for larval movement between sites, suggesting a possibility of genetic connectivity. Future studies incorporating genomic data and larger sample sizes would help clarify environmental and genetic contributions to expression profiles.

Functional profiles also exhibited distinct variations both between holobiont components within the same locality and across locations, highlighting the influence of local environmental conditions on microbial distribution, abundance, and function [47–49]. Despite current limitations, these findings provide a valuable first step toward understanding the transcriptomic dynamics of the *L. santolla* holobiont and lay the groundwork for future studies at broader spatial and temporal scales.

Gill-associated microbial communities exhibit dynamic responses to environmental changes, reflecting shifts in microbial composition in response to varying abiotic conditions. Studies on Atlantic salmon (*Salmo salar*) have demonstrated that gill microbial diversity can change significantly in response to environmental factors, such as seawater input, independent of genetic strain effects [50]. In *L. santolla*, the gill microbiome exhibited compositional variations between locations, likely influenced by local environmental conditions. Similar patterns have been observed in the gill microbiome of other crustaceans, highlighting the influence of environmental factors on microbial function [15, 51]. Notably, salinity has been identified as a major driver of microbiome dynamics in other host-associated environments. For example, experimental studies on green mud crab (*Scylla paramamosain*) exposed to varying salinity levels demonstrated significant shifts in intestinal microbiome composition, underscoring the role of salinity as a key driver in shaping microbial communities [52]. Although derived from gut microbiota, these findings suggest that osmotic pressure similarly affects gill microbiomes, potentially influencing microbial functionality and host–microbiome interactions across different anatomical niches. Despite slight site-specific differences in microbiome composition, Proteobacteria remained the dominant microbial phylum at both locations, consistent with prior studies on estuarine and marine crustacean gills [31, 47, 53, 54]. Proteobacteria are frequently associated with oxidative processes, responses to stimuli, and defense mechanisms [55], suggesting a conserved role in holobiont function across environmental conditions.

Enrichment analysis highlighted metabolic and immune-related pathways associated with physiological response to environmental conditions. At microbiome level, Ballena samples exhibited enrichment in pathways related to carbon metabolism, citrate cycle (TCA), and other carbon fixation pathways. The enrichment of these pathways suggests that microbial communities in this environment rely on aerobic respiration and carbon cycling to maintain energy production. Similar metabolic shifts have been observed in the gut microbiome of Chinese white shrimp *(Fenneropenaeus chinensis)*, where increased energy metabolism was linked to osmotic fluctuations [56]. In addition, Ballena microbiome showed enrichment in autotrophic/chemosynthetic functions, particularly carbon fixation and metal-binding functions (e.g., *RuBisCO*, *nickel-dependent hydrogenase large subunit*). Redox-associated activities were also observed, suggesting microbial engagement in CO₂ fixation and sulfur compound metabolism detoxification (e.g., *ferredoxin oxidoreductase*, *carbon monoxide dehydrogenase*). Such functions may support detoxification and energy conservation strategies under fluctuating conditions. Comparable mechanisms have been described in fiddler crab gill microbiome, revealing that bacteria use such enzymes to convert host-excreted compounds into microbial biomass to mitigate gas toxicity [15]. Further research is needed to confirm whether these microbial functions contribute directly to *L. santolla* detoxification and nutrient recycling under fluctuating environmental conditions.

Meanwhile, Choiseul microbial communities revealed enrichment in transport-related activities, also indicating active regulation of ion balance and solute flux (e.g., *Na,H/K antiporter P-type*, *vacuolar proton-inorganic pyrophosphatase*, *F₀F₁ ATP synthase subunit alpha*). Additionally, ABC-type transporter functions featured prominently, often associated with compatible solute uptake and ion export. This may reflect microbial response to environmental salinity fluctuations. Previous studies in salinity shifts influence on marine microbiome revealed that Na +/H + antiporters’ activity increased with higher salinity levels, pointing to a potential mechanism for microbial response to hypersaline conditions [57]. Likewise, in hypersaline environments, ABC transporters often mediate the uptake of compatible solutes, facilitating the export of excess ion and contributing to cellular homeostasis in saline conditions [58–60]. Further research is needed to determine whether these microbial responses represent independent responses to local conditions or functional integration with *L. santolla* physiology.

At the host level, Ballena samples showed enrichment in endocytosis and antigen processing and presentation, indicating potential metabolic shifts and immune activation in response to environmental conditions. Similar metabolic adjustments have been observed in Chinese mitten crab (*Eriocheir sinensis*) under saline stress [61]. This pattern was also reflected in the enrichment of genes associated with immune regulation (e.g., *crustin*, *type IIa crustin*), consistent with antimicrobial defense mechanisms common in crustaceans [62]. Their enrichment may indicate an immune response, perhaps to changing microbial loads or response to stress. To clarify the drivers of this response, future studies combining microbial profiling and host immune gene expression could help clarify whether these responses are driven by microbiome shifts or environmental stressors. Enrichment of genes involved in solute and ion transport was also observed (e.g., *sodium-dependent phosphate transporter 2*, *zinc transporter 2*), which may contribute to maintaining ionic and osmotic balance under environmental stress. However, the physiological relevance and regulation of these transporters in *L. santolla*, especially under salinity fluctuations, remain to be investigated.

In Choiseul, hosts exhibited enrichment in signaling pathways such as Rap1 and mTOR. The activation of mTOR pathways might reflect regulatory mechanisms involved in immune responses and metabolic adjustments to environmental fluctuations. Similarly, in red swamp crayfish (*Procambarus clarkii*), exposure to different salinity levels has been associated with elevated energy demands and activation of immune and antioxidant pathways, reflecting physiological adjustments to osmotic stress [63]. Enrichment of GO terms also pointed to ion regulation and energy metabolism, with the upregulation of genes related to transmembrane transport and nitrogen excretion (e.g., *Rh type A ammonium transporter*, *ATP synthase*). These may reflect increased osmoregulatory demands in the Choiseul environment. As demonstrated in previous studies, *RhAG* is involved in ammonium excretion through gills, where it supports both nitrogen waste elimination and osmotic regulation [13, 64]. Therefore, its enrichment may indicate a physiological response to heightened osmoregulatory pressure. However, further research is needed to understand the specific function of *RhAG* in *L. santolla*. Antioxidant mechanisms were also apparent, marked by the expression of redox-balancing enzymes (e.g., *peroxiredoxin*, *thioredoxin-dependent peroxide reductase*, *fumarate hydratase*, *NADH-ubiquinone oxidoreductase*). Previous studies have shown that hypersaline conditions trigger oxidative stress in crustaceans such as oriental river prawn (*Macrobrachium nipponense*) [65]. Similarly, in Asian paddle crab (*Charybdis japonica*), elevated oxidative biomarkers and antioxidant responses were observed under hypersaline exposure, suggesting that salinity stress disrupts redox homeostasis [66]. Determining the relevance of these antioxidant mechanisms under salinity challenges would help determine the physiological relevance of these responses in *L. santolla*.

Overall, the observed differences in host and microbial pathway enrichment between Ballena and Choiseul suggest that environmental conditions influence distinct functional responses within the *L. santolla* holobiont. While hosts from both locations modulate transport and stress-related processes through different molecular pathways, their associated microbial communities exhibited metabolic and regulatory profiles. These findings underscore the functional plasticity of the gill-associated microbiome and provide insights into host transcriptional response to fluctuating marine environments. As global climate change and anthropogenic disturbances continue to shape southern marine systems, understanding how host-microbiome interactions respond to abiotic variation is essential for predicting species resilience. Future studies should expand sampling across additional locations and seasons, increase biological replicates, and integrate multi-omic approaches with environmental monitoring which will further elucidate the mechanisms underlying microbial transcriptome response and host-microbiome interactions in *L. santolla*. Such efforts will be essential to disentangle context-dependent microbial functions and better characterize the ecological roles of microbial communities in sub-Antarctic crustaceans.

## Supplementary Information

Supplementary Material 1: Figure S1 Map of the study area in southern Chile. Sampling sites at Ballena Sound and Choiseul Bay are indicated with blue dots. Both locations are situated on Santa Inés Island of the Strait of Magellan. Figure S2 Relative abundance of bacterial classes within the dominant phyla Proteobacteria, Bacteroidetes, and Verrucomicrobia associated with the gills of Lithodes santolla across two locations. Stacked bar plots represent the relative abundance (%) of bacterial classes within each phylum from samples collected at Ballena Sound and Choiseul Bay. Figure S3 Functional annotation and shared GO terms between *Lithodes santolla* and its microbiome in Ballena Sound (BA) and Choiseul Bay (CH). (A) Circos plot showing the distribution of GO terms across datasets: BA Host (blue), CH Host (red), BA Microbiome (orange), and CH Microbiome (green). GO categories: Biological Process (BP, purple), Cellular Component (CC, cyan), and Molecular Function (MF, yellow). (B–D) Venn diagrams of shared and unique GO terms for (B) BP, (C) CC, and (D) MF. Figure S4 Venn diagram of shared and unique Enzyme Classes (EC) between holobiont components (host and microbiome) of Ballena Sound (BA) and Choiseul Bay (CH). Figure S5 Enriched KEGG pathways (FDR < 0.05) for (A) Host and (B) Microbiome under Ballena and Choiseul conditions. Bubble size represents the significance of pathway enrichment (-log10(FDR)), with larger circles corresponding to higher significance. Colors indicate KEGG pathway categories.Table S1 Morphometric measurements of each individual. Table S2 Physicochemical conditions of sampling sites. Table S3 RNA extraction quality and sequencing read statistics from *Lithodes santolla* samples in Ballena Sound and Choiseul Bay. Table S4 Assembly completeness assessment with BUSCO against eukaryote and prokaryote orthologs datasets. Table S6 Number of predicted ORFs corresponding to different enzyme classes.

Supplementary Material 2: Table S5. Functional analysis of differentially enriched GO terms between Ballena and Choiseul samples.

Supplementary Material 3: Table S7. Enriched KEGG pathways from Ballena and Choiseul samples.

## Data Availability

The raw RNA-Seq datasets used in this study are publicly available at the NCBI Sequence Read Archive under the BioProject PRJNA1254097. Additional processed data is available from the corresponding author upon reasonable request.

## References

[1] Apprill A. Marine animal microbiomes: toward understanding host–microbiome interactions in a changing ocean. Front Mar Sci. 2017;4:222. 10.3389/fmars.2017.00222.

[2] Weiland-Bräuer N. Friends or foes—microbial interactions in nature. Biology (Basel). 2021;10(6):496. 10.3390/biology10060496. 10.3390/biology10060496 PMC8229319 34199553

[3] Akbar S, et al. Understanding host-microbiome-environment interactions: insights from *Daphnia* as a model organism. Sci Total Environ. 2022;808: 152093. 10.1016/j.scitotenv.2021.152093. 34863741 10.1016/j.scitotenv.2021.152093

[4] Sehnal L, et al. Microbiome composition and function in aquatic vertebrates: small organisms making big impacts on aquatic animal health. Front Microbiol. 2021. 10.3389/fmicb.2021.567408. 33776947 10.3389/fmicb.2021.567408 PMC7995652

[5] Hou K, et al. Microbiota in health and diseases. Signal Transduct Target Ther. 2022;7: 135. 10.1038/s41392-022-00974-4. 35461318 10.1038/s41392-022-00974-4 PMC9034083

[6] Dittami SM, et al. A community perspective on the concept of marine holobionts: current status, challenges, and future directions. PeerJ. 2021. 10.7717/peerj.10911. 33665032 10.7717/peerj.10911 PMC7916533

[7] Rosenberg E, Zilber-Rosenberg I. Microbes drive evolution of animals and plants: the hologenome concept. mBio. 2016;7. 10.1128/mbio.01395-15. 10.1128/mBio.01395-15 PMC4817260 27034283

[8] Ribas MP, et al. Improving the assessment of ecosystem and wildlife health: microbiome as an early indicator. Curr Opin Biotechnol. 2023;81: 102923. 36996728 10.1016/j.copbio.2023.102923

[9] Stock W, et al. Human impact on symbioses between aquatic organisms and microbes. Aquat Microb Ecol. 2021;87:113–38.

[10] Chung SS-W, et al. The interplay between host-specificity and habitat-filtering influences sea cucumber microbiota across an environmental gradient of pollution. Environ Microbiome. 2024;19: 74. 10.1186/s40793-024-00620-2. 39397007 10.1186/s40793-024-00620-2 PMC11479550

[11] Sylvain F-É, et al. pH drop impacts differentially skin and gut microbiota of the Amazonian fish tambaqui (*Colossoma macropomum*). Sci Rep. 2016;6:32032. 10.1038/srep32032. 27535789 10.1038/srep32032 PMC4989189

[12] Sylvain F-É, et al. Fish skin and gut microbiomes show contrasting signatures of host species and habitat. Appl Environ Microbiol. 2020;86:e00789-e820. 10.1128/AEM.00789-20. 32503908 10.1128/AEM.00789-20 PMC7414953

[13] Henry RP, et al. Multiple functions of the crustacean gill: osmotic/ionic regulation, acid-base balance, ammonia excretion, and bioaccumulation of toxic metals. Front Physiol. 2012;3:431. 10.3389/fphys.2012.00431. 23162474 10.3389/fphys.2012.00431 PMC3498741

[14] Lucu Č, Towle DW. Na++K+-ATPase in gills of aquatic crustacea. Comp Biochem Physiol A Mol Integr Physiol. 2003;135:195–214. 10.1016/S1095-6433(03)00064-3. 12781821 10.1016/s1095-6433(03)00064-3

[15] Fusi M, et al. Gill-associated bacteria are homogeneously selected in amphibious mangrove crabs to sustain host intertidal adaptation. Microbiome. 2023;11:189. 10.1186/s40168-023-01629-4. 37612775 10.1186/s40168-023-01629-4 PMC10463870

[16] Andrade C, et al. Trophic niche dynamics and diet partitioning of king crab *Lithodes santolla* in Chile’s sub-Antarctic water. Diversity. 2022. 10.3390/d14010056.

[17] Bianchi TS, et al. Fjords as aquatic critical zones (ACZs). Earth-Sci Rev. 2020;203: 103145.

[18] Silva N, Vargas CA. Hypoxia in Chilean Patagonian fjords. Prog Oceanogr. 2014;129:62–74.

[19] Sepúlveda J, Pantoja S, Hughen KA. Sources and distribution of organic matter in northern Patagonia fjords, Chile (∼44–47°S): a multi-tracer approach for carbon cycling assessment. Cont Shelf Res. 2011;31:315–29.

[20] Bénard A, Vavre F, Kremer N. Stress & symbiosis: heads or tails? Front Ecol Evol. 2020. 10.3389/fevo.2020.00167.

[21] Ye Y. Editorial: Nutrition, disease, environmental stress, and microorganisms in crustacean aquaculture. Front Mar Sci. 2022;9:1056109. 10.3389/fmars.2022.1056109.

[22] Lafuente E, et al. Effects of anthropogenic stress on hosts and their microbiomes: treated wastewater alters performance and gut microbiome of a key detritivore (*Asellus aquaticus*). Evol Appl. 2023;16:824–48. 37124094 10.1111/eva.13540 PMC10130563

[23] Horváthová T, et al. Tolerance to environmental pollution in the freshwater crustacean *Asellus aquaticus*: a role for the microbiome. Environ Microbiol Rep. 2024;16: e13252. 38783543 10.1111/1758-2229.13252 PMC11116767

[24] Li J, et al. Editorial: Interaction between marine invertebrates and symbiotic microbes in a changing environment: community structure and ecological functions. Front Mar Sci. 2023. 10.3389/fmars.2022.1128906.

[25] Jiang Y, et al. Metatranscriptomic analysis of diverse microbial communities reveals core metabolic pathways and microbiome-specific functionality. Microbiome. 2016;4:2. 10.1186/s40168-015-0146-x. 26757703 10.1186/s40168-015-0146-x PMC4710996

[26] Ojala T, Kankuri E, Kankainen M. Understanding human health through metatranscriptomics. Trends Mol Med. 2023;29:376–89. 10.1016/j.molmed.2023.02.002. 36842848 10.1016/j.molmed.2023.02.002

[27] Dash HR, Das S. Chapter 4 - Molecular methods for studying microorganisms from atypical environments. In: Gurtler V, Trevors JT, editors. Methods in Microbiology. Academic Press; 2018. p. 89–122.

[28] Theissinger K, et al. How genomics can help biodiversity conservation. Trends Genet. 2023;39:545–59. 10.1016/j.tig.2023.01.005. 36801111 10.1016/j.tig.2023.01.005

[29] Pavey SA, et al. The role of gene expression in ecological speciation. Ann N Y Acad Sci. 2010;1206:110–29. 20860685 10.1111/j.1749-6632.2010.05765.x PMC3066407

[30] Barros I, et al. Metatranscriptomics profile of the gill microbial community during *Bathymodiolus azoricus* aquarium acclimatization at atmospheric pressure. AIMS Microbiol. 2018;4:240–60. 10.3934/microbiol.2018.2.240. 31294213 10.3934/microbiol.2018.2.240 PMC6604929

[31] Bacci G, et al. Species-specific gill’s microbiome of eight crab species with different breathing adaptations. Sci Rep. 2023;13:21033. 10.1038/s41598-023-48308-w. 38030652 10.1038/s41598-023-48308-w PMC10687215

[32] Bolger AM, Lohse M, Usadel B. Trimmomatic: a flexible trimmer for Illumina sequence data. Bioinformatics. 2014;30:2114–20. 10.1093/bioinformatics/btu170. 24695404 10.1093/bioinformatics/btu170 PMC4103590

[33] Kopylova E, Noé L, Touzet H. Sortmerna: fast and accurate filtering of ribosomal RNAs in metatranscriptomic data. Bioinformatics. 2012;28:3211–7. 10.1093/bioinformatics/bts611. 23071270 10.1093/bioinformatics/bts611

[34] Grabherr MG, et al. Full-length transcriptome assembly from RNA-seq data without a reference genome. Nat Biotechnol. 2011;29:644–52. 10.1038/nbt.1883. 21572440 10.1038/nbt.1883 PMC3571712

[35] Langmead B, Salzberg SL. Fast gapped-read alignment with Bowtie 2. Nat Methods. 2012;9:357–9. 10.1038/nmeth.1923. 22388286 10.1038/nmeth.1923 PMC3322381

[36] Huang Y, et al. CD-HIT suite: a web server for clustering and comparing biological sequences. Bioinformatics. 2010;26:680–2. 10.1093/bioinformatics/btq003. 20053844 10.1093/bioinformatics/btq003 PMC2828112

[37] Simão FA, et al. BUSCO: assessing genome assembly and annotation completeness with single-copy orthologs. Bioinformatics. 2015;31:3210–2. 10.1093/bioinformatics/btv351. 26059717 10.1093/bioinformatics/btv351

[38] Buchfink B, Reuter K, Drost H-G. Sensitive protein alignments at tree-of-life scale using DIAMOND. Nat Methods. 2021;18:366–8. 10.1038/s41592-021-01101-x. 33828273 10.1038/s41592-021-01101-x PMC8026399

[39] Huson DH, et al. MEGAN community edition - interactive exploration and analysis of large-scale microbiome sequencing data. PLoS Comput Biol. 2016;12: e1004957. 27327495 10.1371/journal.pcbi.1004957 PMC4915700

[40] McHugh ML. The chi-square test of independence. Biochem Med (Zagreb). 2013;23:143–9. 10.11613/BM.2013.018. 23894860 10.11613/BM.2013.018 PMC3900058

[41] Cantalapiedra CP, et al. eggNOG-mapper v2: functional annotation, orthology assignments, and domain prediction at the metagenomic scale. Mol Biol Evol. 2021;38:5825–9. 10.1093/molbev/msab293. 34597405 10.1093/molbev/msab293 PMC8662613

[42] Patro R, et al. Salmon provides fast and bias-aware quantification of transcript expression. Nat Methods. 2017;14:417–9. 10.1038/nmeth.4197. 28263959 10.1038/nmeth.4197 PMC5600148

[43] Robinson MD, Oshlack A. A scaling normalization method for differential expression analysis of RNA-seq data. Genome Biol. 2010;11:R25. 10.1186/gb-2010-11-3-r25. 20196867 10.1186/gb-2010-11-3-r25 PMC2864565

[44] Love MI, Huber W, Anders S. Moderated estimation of fold change and dispersion for RNA-seq data with DESeq2. Genome Biol. 2014;15:550. 10.1186/s13059-014-0550-8. 25516281 10.1186/s13059-014-0550-8 PMC4302049

[45] Calcagno JA, et al. Larval development of the subantarctic king crabs *Lithodes santolla* and *Paralomis granulosa* reared in the laboratory. Helgol Mar Res. 2004;58:11–4. 10.1007/s10152-003-0157-z.

[46] Anger K, et al. Larval and early juvenile development of *Lithodes santolla* (Molina, 1782) (Decapoda: Anomura: Lithodidae) reared at different temperatures in the laboratory. J Exp Mar Biol Ecol. 2004;306:217–30. 10.1016/J.JEMBE.2004.01.010.

[47] Hou D, et al. Environmental factors shape water microbial community structure and function in shrimp cultural enclosure ecosystems. Front Microbiol. 2017;8: 289116. 10.3389/FMICB.2017.02359. 10.3389/fmicb.2017.02359 PMC5712584 29238333

[48] Wang X, et al. Effects of environmental factors on the distribution of microbial communities across soils and lake sediments in the Hoh Xil Nature Reserve of the Qinghai-Tibetan Plateau. Sci Total Environ. 2022;838: 156148. 10.1016/J.SCITOTENV.2022.156148. 35609688 10.1016/j.scitotenv.2022.156148

[49] Handy RD, et al. The microbiomes of wildlife and chemical pollution: status, knowledge gaps and challenges. Curr Opin Toxicol. 2023;36: 100428. 10.1016/J.COTOX.2023.100428.

[50] Quezada-Rodriguez PR, et al. Assessment of gill microbiome of two strains of Atlantic salmon reared in flowthrough and recirculation hatcheries and following seawater transfer. Aquaculture. 2024;580: 740322. 10.1016/J.AQUACULTURE.2023.740322.

[51] Garuglieri E, et al. Morphological characteristics and abundance of prokaryotes associated with gills in mangrove brachyuran crabs living along a tidal gradient. PLoS One. 2022;17: e0266977. 10.1371/JOURNAL.PONE.0266977. 35421185 10.1371/journal.pone.0266977 PMC9009686

[52] Niu M, et al. Response of intestinal microbiota to saline-alkaline water in mud crab (*Scylla paramamosain*) based on multiple low salinity culture modes. Front Mar Sci. 2023;10: 1153326. 10.3389/FMARS.2023.1153326.

[53] Zhang M, et al. Symbiotic bacteria in gills and guts of Chinese mitten crab (*Eriocheir sinensis*) differ from the free-living bacteria in water. PLoS One. 2016;11: e0148135. 10.1371/JOURNAL.PONE.0148135. 26820139 10.1371/journal.pone.0148135 PMC4731060

[54] Fusi M, et al. Gill-associated bacteria are homogeneously selected in amphibious mangrove crabs to sustain host intertidal adaptation. Microbiome. 2023;11:1–21. 10.1186/S40168-023-01629-4. 37612775 10.1186/s40168-023-01629-4 PMC10463870

[55] Zhou Z, et al. Genome diversification in globally distributed novel marine *Proteobacteria* is linked to environmental adaptation. ISME J. 2020;14:2060–77. 10.1038/S41396-020-0669-4. 32393808 10.1038/s41396-020-0669-4 PMC7367891

[56] Tian C, et al. Integrated analysis of the intestinal microbiota and transcriptome of Fenneropenaeus chinensis response to low-salinity stress. Biology (Basel). 2023;12:1502. 10.3390/BIOLOGY12121502/S1. 38132328 10.3390/biology12121502 PMC10741032

[57] Fortunato CS, Crump BC. Microbial gene abundance and expression patterns across a river to ocean salinity gradient. PLoS One. 2015;10: e0140578. 26536246 10.1371/journal.pone.0140578 PMC4633275

[58] Rees DC, Johnson E, Lewinson O. ABC transporters: the power to change. Nat Rev Mol Cell Biol. 2009;10:218–27. 10.1038/nrm2646. 19234479 10.1038/nrm2646 PMC2830722

[59] Pal S, et al. ABC-type salt tolerance transporter genes are abundant and mutually shared among the microorganisms of the hypersaline Sambhar Lake. Extremophiles. 2025;29:14. 10.1007/s00792-025-01378-2. 39873828 10.1007/s00792-025-01378-2

[60] Mehta P, et al. Culture-independent exploration of the hypersaline ecosystem indicates the environment-specific microbiome evolution. Front Microbiol. 2021. 10.3389/fmicb.2021.686549. 34777269 10.3389/fmicb.2021.686549 PMC8581802

[61] Zhang D, et al. Comparative transcriptome analysis of *Eriocheir japonica sinensis* response to environmental salinity. PLoS One. 2018;13: e0203280. 10.1371/JOURNAL.PONE.0203280. 30192896 10.1371/journal.pone.0203280 PMC6128516

[62] Zhang W, et al. Discovery and characterization of a new crustin antimicrobial peptide from *Amphibalanus amphitrite*. Pharmaceutics. 2022. 10.3390/pharmaceutics14020413. 35214145 10.3390/pharmaceutics14020413 PMC8877177

[63] Luo L, et al. Effects of different salinity stress on the transcriptomic responses of freshwater crayfish (*Procambarus clarkii*, Girard, 1852). Biology. 2024. 10.3390/biology13070530. 39056722 10.3390/biology13070530 PMC11273973

[64] Weihrauch D, Wilkie MP, Walsh PJ. Ammonia and urea transporters in gills of fish and aquatic crustaceans. J Exp Biol. 2009;212:1716–30. 10.1242/jeb.024851. 19448081 10.1242/jeb.024851

[65] Xue C, et al. Transcriptome analysis to study the molecular response in the gill and hepatopancreas tissues of *Macrobrachium nipponense* to salinity acclimation. Front Physiol. 2022. 10.3389/fphys.2022.926885. 35694393 10.3389/fphys.2022.926885 PMC9176394

[66] Shui B, et al. Salinity fluctuation on the genetic regulatory mechanisms of the crustacean, *Charybdis japonica*. Front Mar Sci. 2022. 10.3389/fmars.2022.870891.

