# Supplementary material for: Environmental variability drives functional plasticity in the gill-associated microbiome of Lithodes santolla: a meta-transcriptomic perspective
Source: Microbiome. 2025 Oct 1;13:197. doi: 10.1186/s40168-025-02215-6 (PMC12487024; doi:10.1186/s40168-025-02215-6)
Supplement: Supplementary file 2 — Supplementary Material 1: Figure S1 Map of the study area in southern Chile. Sampling sites at Ballena Sound and Choiseul Bay are indicated with blue dots. Both locations are situated on Santa Inés Island of the Strait of Magellan. Figure S2 Relative abundance of bacterial classes within the dominant phyla Proteobacteria, Bacteroidetes, and Verrucomicrobia associated with the gills of Lithodes santolla across two locations. Stacked bar plots represent the relative abundance (%) of bacterial classes within each phylum from samples collected at Ballena Sound and Choiseul Bay. Figure S3 Functional annotation and shared GO terms between Lithodes santolla and its microbiome in Ballena Sound (BA) and Choiseul Bay (CH). (A) Circos plot showing the distribution of GO terms across datasets: BA Host (blue), CH Host (red), BA Microbiome (orange), and CH Microbiome (green). GO categories: Biological Process (BP, purple), Cellular Component (CC, cyan), and Molecular Function (MF, yellow). (B–D) Venn diagrams of shared and unique GO terms for (B) BP, (C) CC, and (D) MF. Figure S4 Venn diagram of shared and unique Enzyme Classes (EC) between holobiont components (host and microbiome) of Ballena Sound (BA) and Choiseul Bay (CH). Figure S5 Enriched KEGG pathways (FDR < 0.05) for (A) Host and (B) Microbiome under Ballena and Choiseul conditions. Bubble size represents the significance of pathway enrichment (-log10(FDR)), with larger circles corresponding to higher significance. Colors indicate KEGG pathway categories.Table S1 Morphometric measurements of each individual. Table S2 Physicochemical conditions of sampling sites. Table S3 RNA extraction quality and sequencing read statistics from Lithodes santolla samples in Ballena Sound and Choiseul Bay. Table S4 Assembly completeness assessment with BUSCO against eukaryote and prokaryote orthologs datasets. Table S6 Number of predicted ORFs corresponding to different enzyme classes. [file 40168_2025_2215_MOESM1_ESM.docx]

# Environmental Variability Drives Functional Plasticity in the Gill-Associated Microbiome of *Lithodes santolla*: A Meta-Transcriptomic Perspective

Alexandra Brante ^1,2^, Paulina Bustos ^3,4^, Claudio Ortega-Muñoz ^1,2^, Eliana Paola Acuña Gómez ^2^, Vicenzo Brante ^3^ and Rodolfo Farlora ^4,5^ *

*Corresponding author: R. Farlora, Laboratorio de Biotecnología Acuática y Genómica Reproductiva (LABYGER), Facultad de Ciencias, Universidad de Valparaíso, Gran Bretaña 1111, Playa Ancha, Valparaíso 2360102, Chile. Phone Number: +56322508200. E-mail: [rodolfo.farlora@uv.cl](mailto:rodolfo.farlora@uv.cl) ORCID ID: <https://orcid.org/0000-0002-0048-1102>.

1. Programa de Magíster en Ciencias Biológicas mención Biodiversidad y Conservación, Instituto de Biología, Facultad de Ciencias, Universidad de Valparaíso.

2. Centro de Estudios del Cuaternario de Fuego - Patagonia y Antártica (CEQUA), Punta Arenas, Chile.

3. Laboratorio de Microbiología Integrativa e Innovación Tecnológica (MIIB-Lab), Instituto de Biología, Facultad de Ciencias, Universidad de Valparaíso, Valparaíso, Chile

4. Centro de Investigación y Gestión de Recursos Naturales (CIGREN), Universidad de Valparaíso, Valparaíso, Chile.

5. Laboratorio de Biotecnología Acuática y Genómica Reproductiva (LABYGER), Instituto de Biología, Facultad de Ciencias, Universidad de Valparaíso, Valparaíso, Chile.

Supplementary Information


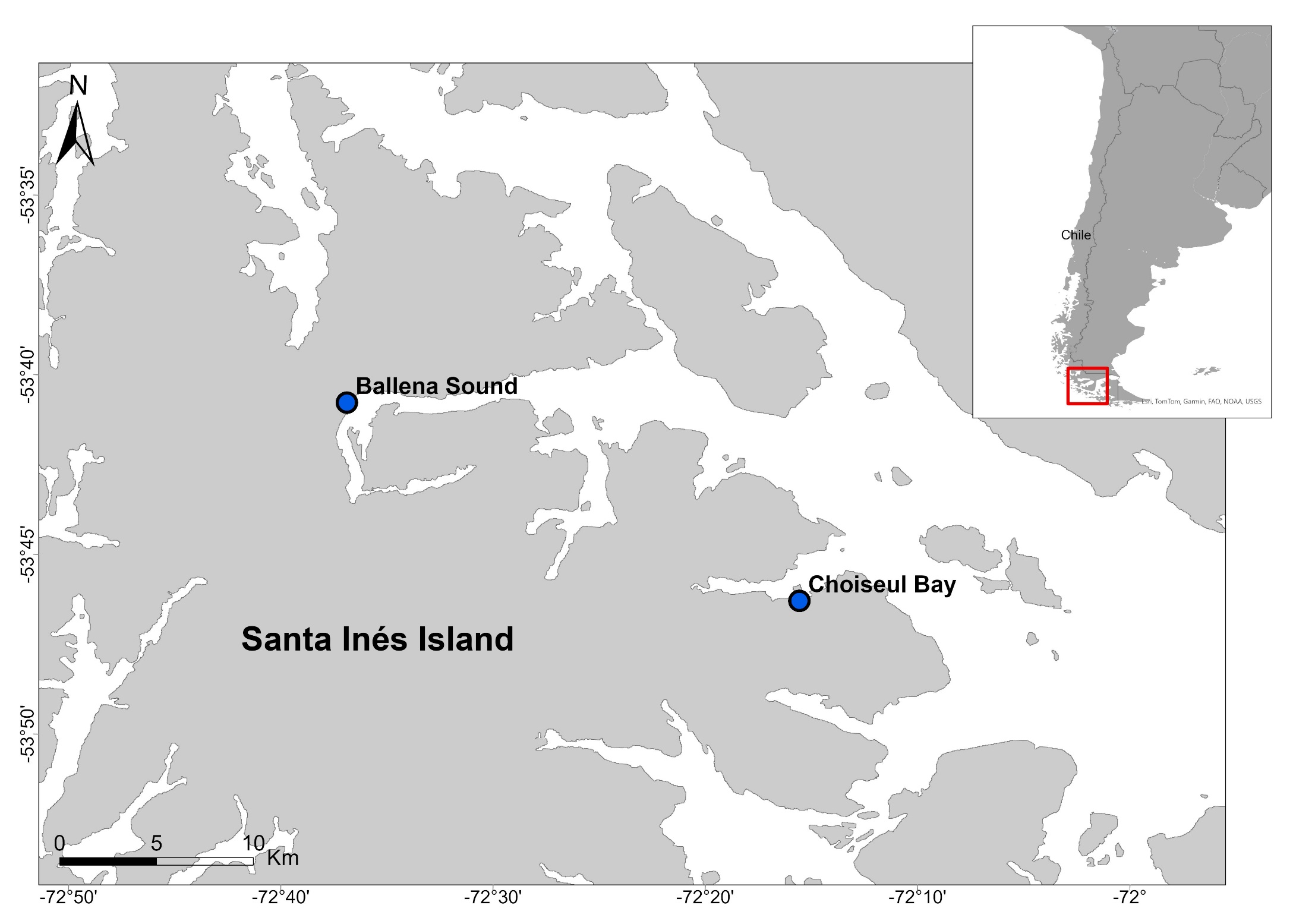


Figure S1 Map of the study area in southern Chile. Sampling sites at Ballena Sound and Choiseul Bay are indicated with blue dots. Both locations are situated on Santa Inés Island of the Strait of Magellan.


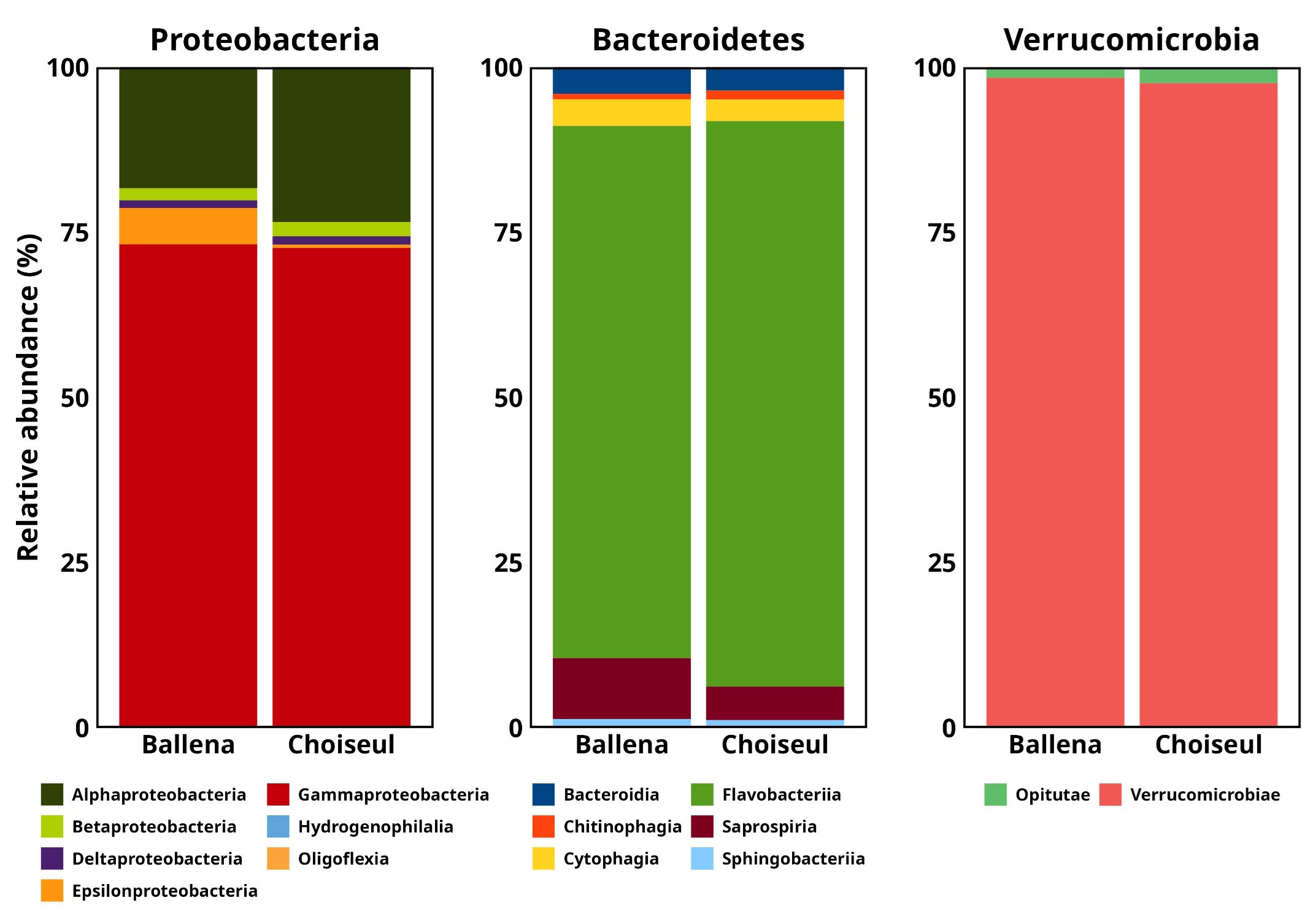


Figure S2 Relative abundance of bacterial classes within the dominant phyla Proteobacteria, Bacteroidetes, and Verrucomicrobia associated with the gills of Lithodes santolla across two locations. Stacked bar plots represent the relative abundance (%) of bacterial classes within each phylum from samples collected at Ballena Sound and Choiseul Bay.


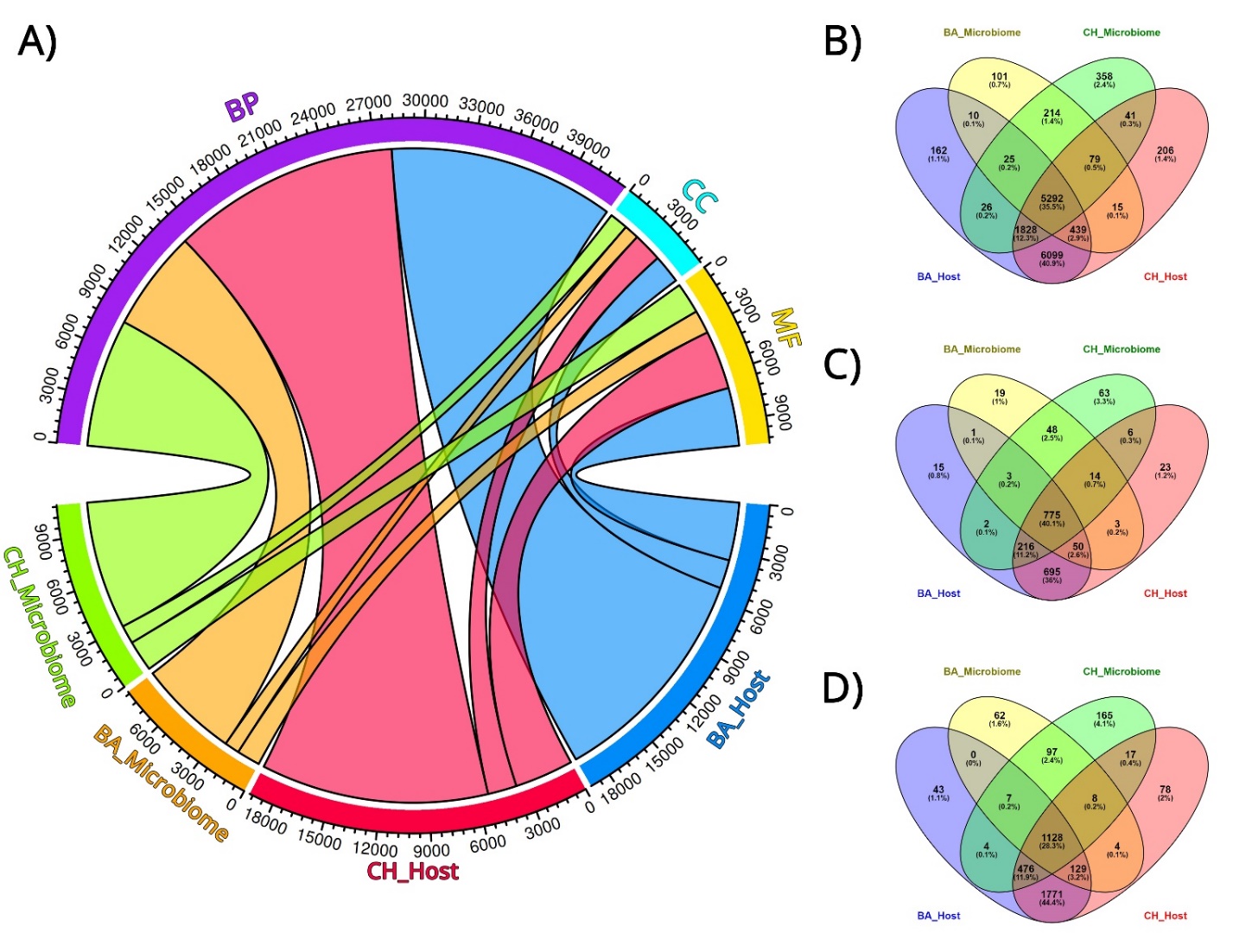


Figure S3 Functional annotation and shared GO terms between *Lithodes santolla* and its microbiome in Ballena Sound (BA) and Choiseul Bay (CH). (A) Circos plot showing the distribution of GO terms across datasets: BA Host (blue), CH Host (red), BA Microbiome (orange), and CH Microbiome (green). GO categories: Biological Process (BP, purple), Cellular Component (CC, cyan), and Molecular Function (MF, yellow). (B–D) Venn diagrams of shared and unique GO terms for (B) BP, (C) CC, and (D) MF.


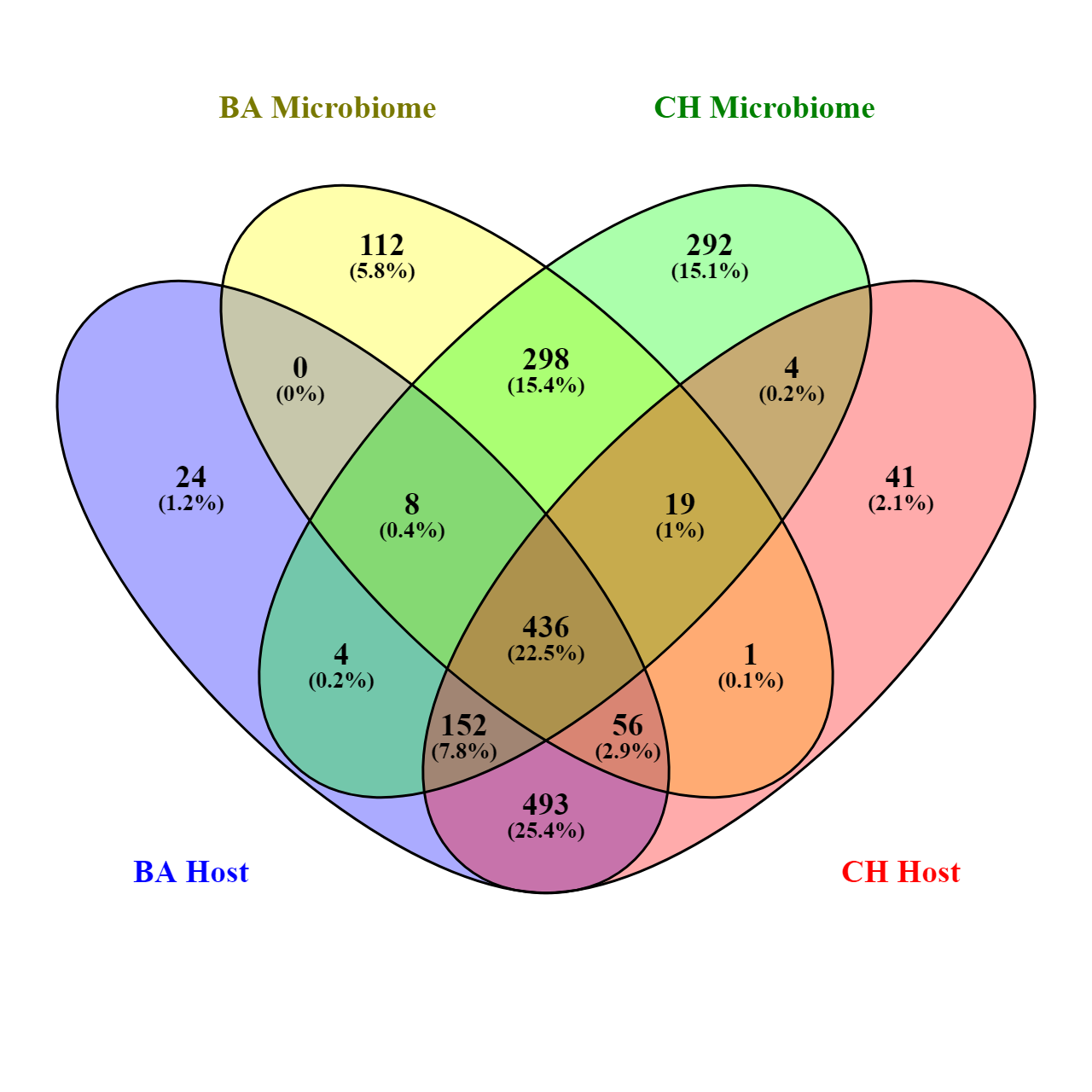


**Figure S4** Venn diagram of shared and unique Enzyme Classes (EC) between holobiont components (host and microbiome) of Ballena Sound (BA) and Choiseul Bay (CH).


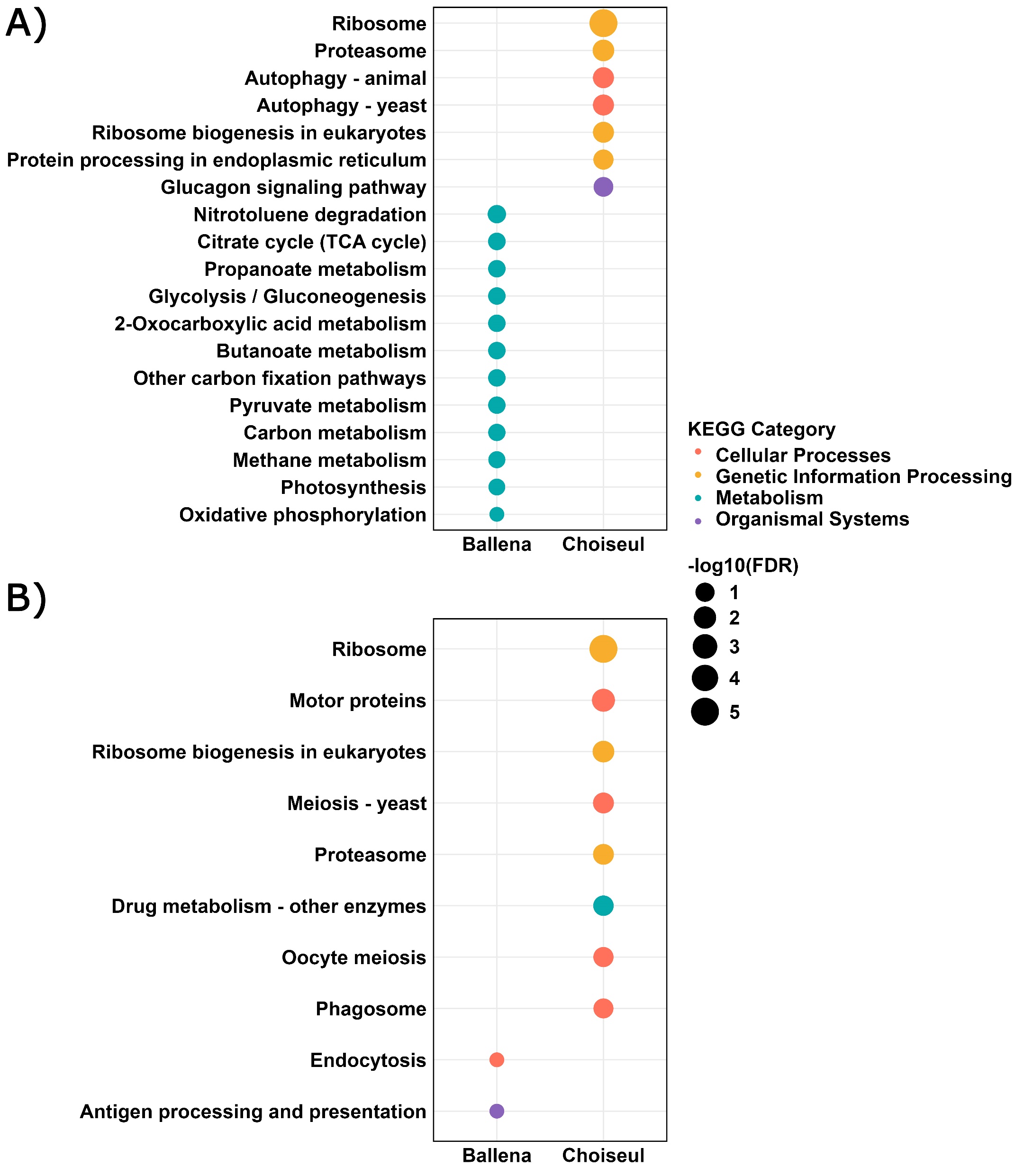


Figure S5 Enriched KEGG pathways (FDR < 0.05) for (A) Host and (B) Microbiome under Ballena and Choiseul conditions. Bubble size represents the significance of pathway enrichment (-log10(FDR)), with larger circles corresponding to higher significance. Colors indicate KEGG pathway categories.

**Table S1** Morphometric measurements of each individual.

| Sampling site | Sample ID | Sex | CL (mm) | CW (mm) | Body Weight (g) |
| --- | --- | --- | --- | --- | --- |
| Ballena Sound | CM1223 | Male | 111.0 | 85.0 | 385 |
|  | CM1231 | Male | 74.4 | 62.4 | 185 |
|  | CM1241 | Male | 89.5 | 80.6 | 323 |
|  | CM1264 | Male | 85.5 | 69.7 | 238 |
|  | CM1272 | Male | 86.5 | 73.3 | 299 |
| Choiseul Bay | CM1101 | Male | 99.3 | 80.0 | 248 |
|  | CM1112 | Male | 91.1 | 75.2 | 227 |
|  | CM1125 | Male | 88.4 | 68.2 | 212 |
|  | CM1155 | Male | 75.5 | 62.0 | 138 |
|  | CM1195 | Male | 66.0 | 55.0 | 96.0 |

**Table S2** Physicochemical conditions of sampling sites.

| Sampling site | *pH* | T (°C) | Dissolved Oxygen (mg/L) | Oxygen Saturation (%) | Salinity (PSU) |
| --- | --- | --- | --- | --- | --- |
| Ballena Sound | 8.03 | 7.63 | 9.86 | 92.31 | 24.66 |
| Choiseul  Bay | 8.03 | 8.74 | 11.2 | 95.36 | 29.21 |

**Table S3** RNA extraction quality and sequencing read statistics from *Lithodes santolla* samples in Ballena Sound and Choiseul Bay.

| Sampling site | Sample | Total RNA (μg) | A260/280 | IQ | Raw reads | Clean reads |
| --- | --- | --- | --- | --- | --- | --- |
| Ballena Sound | CM1223 | 29.2800 | 2.15 | 7.8 | 39,476,492 | 30,799,216 |
|  | CM1231 | 17.4080 | 2.14 | 8.4 | 45,409,316 | 33,802,624 |
|  | CM1241 | 24.8000 | 2.11 | 8.7 | 41,153,724 | 32,311,992 |
|  | CM1264 | 26.8480 | 2.14 | 8.3 | 43,781,506 | 32,849,162 |
|  | CM1272 | 31.0400 | 2.09 | 8.0 | 42,973,504 | 33,179,344 |
| Choiseul Bay | CM1101 | 38.3040 | 2.08 | 8.3 | 48,214,986 | 36,559,454 |
|  | CM1112 | 5.4400 | 2.14 | 8.2 | 42,974,042 | 31,859,034 |
|  | CM1125 | 20.3200 | 2.14 | 8.5 | 47,894,734 | 35,417,990 |
|  | CM1155 | 17.7920 | 2.15 | 8.1 | 47,906,876 | 37,547,254 |
|  | CM1195 | 19.2640 | 2.06 | 7.8 | 39,852,918 | 28,378,262 |

**Table S4** Assembly completeness assessment with BUSCO against eukaryote and prokaryote orthologs datasets.

| Assembly | Reference dataset | Complete | Single copy | Duplicate | Fragmented | Missing |
| --- | --- | --- | --- | --- | --- | --- |
| Global | Arthropoda | 94.1 | 63.9 | 30.2 | 3.4 | 2.5 |
|  | Eukaryote | 99.2 | 66.3 | 32.9 | 0.8 | 0.0 |
|  | Prokaryote | 65.3 | 17.7 | 47.6 | 10.9 | 23.8 |
| Ballena | Arthropoda | 92.5 | 70.0 | 22.5 | 4.6 | 2.9 |
|  | Eukaryote | 96.9 | 71.4 | 25.5 | 3.1 | 0.0 |
|  | Prokaryote | 63.0 | 22.8 | 40.2 | 9.0 | 28.0 |
| Choiseul | Arthropoda | 92.6 | 69.0 | 23.6 | 4.4 | 3.0 |
|  | Eukaryote | 96.1 | 74.5 | 21.6 | 3.5 | 0.4 |
|  | Prokaryote | 63.4 | 17.4 | 46.0 | 10.9 | 25.7 |

**Table S6** Number of predicted ORFs corresponding to different enzyme classes

| Enzyme class | Ballena Host | Ballena microbiome | Choiseul Host | Choiseul microbiome |
| --- | --- | --- | --- | --- |
| EC1: Oxidoreductases | 1,138 | 850 | 1,200 | 1,577 |
| EC2: Transferases | 4,032 | 1,201 | 4,049 | 2,824 |
| EC3: Hydrolases | 3,009 | 822 | 3,183 | 1,564 |
| EC4: Lyases | 373 | 254 | 405 | 497 |
| EC5: Isomerases | 265 | 194 | 247 | 397 |
| EC6: Ligases | 353 | 331 | 347 | 692 |
| Total ORFs | 9,170 | 3,652 | 9,341 | 7,551 |
| Percentage (%) | 30.9 | 12.3 | 31.4 | 25.4 |
